# Exercise-induced anaphylaxis: A clinical view

**DOI:** 10.1186/1824-7288-38-43

**Published:** 2012-09-14

**Authors:** Carlotta Povesi Dascola, Carlo Caffarelli

**Affiliations:** 1 Clinica Pediatrica, Dipartimento di Medicina Clinica e Sperimentale, Azienda Ospedaliera-Universitaria di Parma, Università degli Studi di Parma, Via Gramsci 14, Parma, Italy

**Keywords:** Physical exercise, Food allergy, Exercise-induced anaphylaxis, Exercise-induced bronchocostriction, Urticaria, Anaphylaxis

## Abstract

Exercise-induced anaphylaxis (EIA) is a distinct form of physical allergy. The development of anaphylaxis during exertion often requires the concomitant exposure to triggering factors such as intake of foods (food dependent exercise-induced anaphylaxis) or drugs prior to exercise, extreme environmental conditions. EIA is a rare, but serious disorder, which is often undetected or inadequately treated. This article summarizes current evidences on pathophysiology, diagnosis and management. We reviewed recent advances in factors triggering the release of mediators from mast cells which seems to play a pathogenetic role. A correct diagnosis is essential to avoid unnecessary restricted diet, to allow physical activity in subjects with EIA dependent from triggering factors such as food, and to manage attacks. An algorithm for diagnosing EIA based on medical history, IgE tests and exercise challenge test has been provided. In the long-term management of EIA, there is a need for educating patients and care-givers to avoid exposure to precipitating factors and to recognize and treat episodes. Future researches on existing questions are discussed.

## Introduction

Physical exercise may provoke the onset of clinical symptoms that are usually due to an allergic reaction. Distinct forms of recognized physical allergies are exercise-induced anaphylaxis (EIA), cholinergic urticaria, exercise-induced bronchospasm and rhinitis.

Anaphylaxis triggered by physical exertion is a serious disorder which is often undetected or inadequately treated. EIA is rare, with a prevalence of 0.048% in a survey of 76.229 adolescents, aged 13–15 years [1]. Up to 9% of children referred to a tertiary allergy center for anaphylaxis suffer from EIA [2]. EIA affects subjects at any time during lifespan and age of onset varies from 4 to 74 years [3,4]. Concomitant factors may be required to develop anaphylaxis during exertion. In 30% [1] - 50% of cases [3], EIA occurs only when the subject ingests a particular food before exercise, known as specific food dependent exercise-induced anaphylaxis (FDEIA) [5] or a meal (non-specific FDEIA) prior to exercise [6]. Angioedema and oral allergy syndrome have been reported in an 8 years-old boy who ingested tomatoes after physical exercise [7]. Exercise or food alone do not elicit symptoms. Maulitz et al. [5] described the first case of shellfish dependent EIA in 1979. Afterwards, many different types of foods have been reported to predispose the development of EIA. They include celery [8], wheat [9-11], shellfish [12], grapes [11], nuts [13], peaches [13], eggs [14,15], oranges [16], apples [17], hazelnuts [18], chees [19], cabbage [3], chestnuts [20], rice [20], tomatoes [20], cuttlefish [21], pistachios [22], cow’s milk [23], corn, paprika, mustard, barley [24], onions [25], peanuts [15,26], fish [27], snails [28], pork [29], beef [29], chicken/turkey [30], mushrooms [31], buckwheat [32], alcohol [3]. Some patients are sensitive to more than one type of food [20]. In some patients with FDEIA, aspirin intake [33], cold [34] or warm environment [35] are required for developing the reaction. FDEIA may occur only when two types of foods are simultaneously ingested before exercise [29,36]. Moreover, during cow’s milk desensitization, children may have cow’s milk dependent EIA episodes [37]. In many patients with EIA, food intake is not related to EIA occurrence but other precipitating factors have been reported. They are drugs [3,5,8,38], cold or warm temperature, humidity [3,38-40], menstrual cycle [11], dental amalgam [40], pollen season [3], ingestion of dust mites contained in contaminated foods [41]. A family history of EIA has been associated with the HLA A3B8DR3 [42] or with a possible autosomal dominant inheritance [43]. Subjects with EIA or FDEIA [44] are often atopic. These findings might suggest a potential genetic origin for EIA.

### Pathophysiology

In EIA, the release of vasoactive mediators from mast cells may play a pathogenetic role. This has been observed in skin biopsies [45] and it has been confirmed by findings of increased serum histamine [45-47] and tryptase [48] levels in patients with EIA after exercise. Release of mast cell mediators may result in vascular leakage, inflammatory cell recruitment and occurrence of anaphylaxis [49]. The mast cell degranulation may be mediated by IgE antibodies. However, in patients with EIA, serum IgE antibodies are usually normal in patients who do not suffer from allergic diseases [50]. Other triggering factors may be lactate or creatinine phosphokinase [51]. Overall, it remains unclear which factors trigger mast cell degranulation. In patients with FDEIA, the ingestion of the offending food alone does not provoke clinical hypersensitivity reactions, even if IgE antibodies against the causative food allergens are usually detected by skin prick tests or in the serum. Non mutually exclusive explanations have been provided for the loss of tolerance to food during exercise [52]. Some of them are sustained by clinical findings. First, the administration of sodium bicarbonate before physical activity prevents occurrence of symptoms in patients with FDEIA [53]. Therefore it has been suggested that pH modifications might elicit the onset of anaphylaxis. Along this line, it has been reported that after physical activity, pH decreases both in serum and in muscle [54,55]. Furthermore, a reduced pH enhances mast cell degranulation [56]. Second, aspirin appears to induce anaphylactic reaction to wheat by increasing gastrointestinal permeability. This is suggested by the fact that in patients with wheat dependent EIA, both wheat-exercise and wheat-aspirin challenges provoked an increased gliadin absorption and allergic symptoms. Higher serum gliadin levels may cause degranulation of mast cell with onset of anaphylaxis [57]. Small intestinal permeability is increased by exercise [58]. However, it has been shown that serum gladin levels are similarly increased during combination wheat-exercise test in subjects with FDEIA and in controls [56], ruling out a role for increased gastrointestinal permeability.

Further hypotheses have been offered. Mucosal mast cells and connective tissue mast cells differ in release of histamine and leukotrienes [59,60]. Exposure to allergenic foods of gut-specific mast cells seems to lead to tolerate the food. During exercise, there is a redistribution of blood flow from the gut to skin or skeletal muscle where mast cells may be more responsive to the causative food. Consequently, a greater exposure of the connective tissue mast cells to allergenic foods may result in an increased release of mediators and development of EIA [52]. Another explanation has been provided for wheat dependent EIA. Tissue transglutaminase (tTG) are activated by IL-6 produced by skeletal muscle in response to exercise. Pepsin digestion of wheat allergens including omega-5 gliadin, the main wheat allergen in FDEIA [61], forms peptides that are cross-linked by tTG into high-molecular-weight complexes which strongly bind to IgE. It has been suggested that these allergen complexes may elicit anaphylactic reactions in patients with wheat dependent EIA [62].

The autonomic system may also play a role in FDEIA since an increased activity of the parasympathetic nervous system and a reduced activity of the sympathetic nervous system have been reported [14]. Lastly, an increased histamine release from basophils of FDEIA patients has been found at elevated osmolality levels [63,64]. Therefore, serum hyperosmolality might increase basophil releasability after food allergen intake [65]. However, such osmolality levels are not reached in plasma during physical activity [64].

### Clinical presentation

EIA has been associated with both strenuous and mild, especially in children, physical activities [66]. The intensity of the exercise and amount of food ingested may provoke different symptoms from subject to subject and in the same subject [66,67]. Any kind of exercise may potentially induce the reaction. Walking, jogging, running, aerobics, dancing, swimming, cycling, horse riding, tennis, basketball, soccer, skiing, raking leaves have been reported [3,68,69]. Symptoms may appear during warm-up, maintenance or cool-down phases. Patients do not always experience symptoms every time they practice physical activity.

The reaction often occurs within 30 min from the onset of physical exertion. However, Oyefaras BL et al. [70] described a patient who developed wheat-dependent EIA, 5 h after exercise. EIA symptoms may be serious and fatalities have been sporadically reported in adults [32,71,72]. Symptoms of EIA are similar to those of anaphylactic reactions caused by allergens such as foods or hymenoptera venom. In the beginning, children commonly present pruritus or tingling in the extremities followed by generalized pruritus and warmth, urticaria-angioedema and flushing. Among respiratory symptoms, cough, chest thightness, wheezing with impaired spirometric values [73] appear in about 50% of cases while rhinitis, hoarseness and laryngeal dyspnea are less frequent [44]. In 25-30% of cases, sweating, gastrointestinal complaints (frequently vomiting), headache, faintness, collapse and syncope, may occur [44]. Some patients experience late symptoms which occur after 24–48 h (biphasic anaphylaxis). Natural history of EIA is not well known. A reduction in the number of attacks with time has been reported [69], but recovery is unlikely.

### Diagnosis

A correct diagnosis is essential to avoid unnecessary restricted diet, to allow physical activity in subjects with EIA dependent from triggering factors such as food, and to manage attacks. Careful medical history is critical to identify the events that precipitate the reaction and involved cofactors. Generally, history of food reactions is unreliable [74]. Also in FDEIA, history may only suggest foods whose intake is a prerequisite for the development of the reaction [75]. Skin prick tests and/or circulating IgE antibodies for all foods ingested before physical activity are helpful for identifying the offending food. In our experience, in specific FDEIA, IgE test results were positive for the causative food, while non-specific FDEIA or in EIA, IgE test were negative.

**Figure 1:**
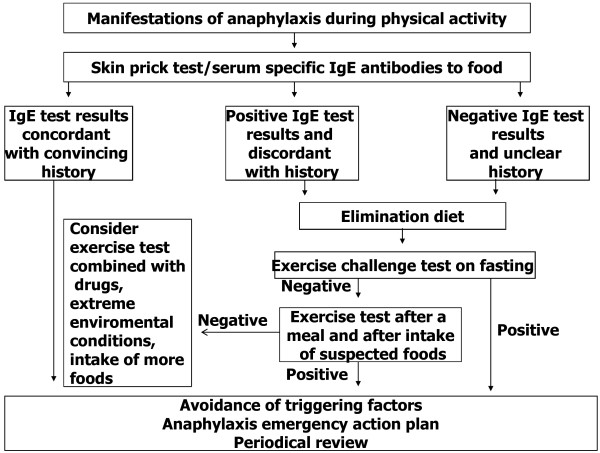
Diagnosis of EIA.

Open challenge test is the definitive mean to ascertain the diagnosis and it is required when medical history and IgE test results are unclear or discordant (Figure 1). The challenge test should be performed under medical supervision with accessibility to equipment and support for managing anaphylactic reactions. When symptoms develop, blood pressure should be monitored [76] and treatment promptly given. In children, the exercise challenge test may consist in performing an exertion continuously for at least 6 min to reach 80% of maximum heart frequency predicted for age, following the protocol for exercise-induced broncocostriction [20,73,75,77]. In children, free running is preferred to tapis roulant or cycle ergometer because it is more easily accepted and performed. Lung function should be measured at baseline, at the end of exercise and then every 3 min for 3 times [73]. The exercise challenge in adults is usually performed according to the protocol for ischemic heart disease [78,79]. Exercise test is performed on fasting to identify subjects with EIA. When it is negative, after successful elimination of suspected foods, a combination food-exercise challenge test is required. Food challenge alone is rarely necessary. The exercise test should be performed 1 h after the ingestion of each of the suspected foods to ascertain specific FDEIA and 1 h after a meal to diagnose non-specific FDEIA. The combined food-exercise test can usually identify the triggering foods. However, a negative exercise challenge test does not exclude the diagnosis of EIA since it is negative in about 30% of cases [11,80]. This is partly explained by the fact that in some cases the exposure to triggering factors such extreme environments [34,35] are necessary to elicit the reaction and they cannot be reproduced in the laboratory. A double-blind placebo controlled combined food-exercise test may be required when symptoms are unusual or ambiguous [81].

The differential diagnosis of childhood EIA with other allergies induced by exertion is challenging. Cholinergic urticaria occurs with small wheals (diameter 2–4 mm) and a large erythema. These lesions frequently arise in the upper part of the chest and subsequently spread to the whole body [82]. A single lesion often resolves in 15–20 min, even if the episode may last 2–4 h. This type of urticaria is triggered by the rise in core body temperature and it is due to both active mechanisms (exercise, stress, fever) and passive (hot shower). Manifestations of EIA (bronchospasm, angioedema, hypotension) may appear in patients with cholinergic urticaria. In these cases, cholinergic urticaria is easily differentiated from EIA, since symptoms are induced not only by physical exertion but also by an increase in body temperature.

Exercise-induced bronchoconstriction is characterized by asthma symptoms or cough [83] that usually occur within 10–15 min after physical activity, rarely during exercise. Symptoms resolve in a variable time within an hour after the end of the exercise. Exercise-induced bronchoconstriction is experienced by most asthmatic children and is not associated with anaphylactic symptoms or food intake. The diagnostic value of medical history [84] in identifying individuals suffering from exercise-induced bronchoconstriction is poor. Hallstrand et al. [85] found that 12% of children with a history of exercise-induced bronchoconstriction and 8.7% of those without history had positive exercise test results. Therefore, the diagnosis of exercise-induced bronchoconstriction should be based on exercise challenge test results. [85]. A positive exercise test is defined as a maximum percentage fall in FEV1 ≥15% from the baseline [77]. Exercise test may be influenced by the type of exercise, environmental conditions, hour of the day, and the health of the child (infections, allergies, drugs).

The following disorders also need to be considered as alternative diagnosis when symptoms occur during exercise: hyperventilation syndrome, panic or vasovagal attacks, vocal cord dysfunction. Hereditary angioedema attacks may occasionally be associated with exercise but they are not caused by food intake and occur also at rest. In cardiac diseases or in neurologic disorders, collapse may occur during physical activity but are not associated with anaphylactic symptoms. Patients with cold-urticaria may develop symptoms of EIA when they practice exercise in a cold environment. Non exercise-dependent food allergy is excluded by a negative IgE test results to a previously tolerated food. In some instances, oral food challenge test is necessary.

### Management

Patients with EIA and their families should be educated to prevent further attacks by avoiding relevant triggers. In children with FDEIA, physical activity is permitted only after at least 4 h from the ingestion of the culprit food [1]. The food should be avoided also for 1 h after exercise [29]. Participation of children with FDEIA in physical activity should be encouraged since it has a positive effect on their physical and social well-being. It has been sporadically reported that some patients respond to prophylactic treatment [46] with sodium cromoglycate [86,87], H2-antihistamines [87-89], leukotriene modifiers [89], steroids [36] or sodium bicarbonate [53]. However, there is not sufficient evidence to routinely recommend preventive drugs. Another point is that patients and care-givers should be instructed to recognize and treat episodes since parents are often unable to schedule exertion in children. A personalized, written anaphylaxis emergency action plan should be given. Patients or care-givers must always keep self-injectable epinephrine available and should be educated on how to use it. Every year, the action plan as well as the practical use of epinephrine should be reviewed. All at-risk patients should never exercise alone, but always with a person who has been trained in treating anaphylactic reactions. Patients should be able to recognize prodromal symptoms and anaphylactic manifestations. At the first occurrence of symptoms, exertion should be terminated. The subject should be put in the supine position [90] and self-injectable epinephrine followed by H2-antihistamines and steroids should be promptly administered. The supine patient should be quickly transported to the hospital.

## Conclusions

EIA represents a growing recognized worldwide health problem. Further studies are necessary to elucidate many existing questions. Hypotheses on EIA pathophysiology should be sustained by clinical observations. Method for exercise challenge test that is considered the gold-standard for diagnosing EIA, needs to be standardized. Finally, if the subjects who benefit from preventive treatment are identified, significant improvement in the health and quality of life of patients with EIA and their families are likely.

## Abbreviations

EIA: Exercise-induced anaphylaxis; FDEIA: Food dependent exercise-induced anaphylaxis.

## Competing interests

The authors declare that they have no competing interests.

## Authors’ contribution

All authors declare to have equally contributed to choose the subject, writing of the paper, and reviewing the literature data. Both authors read and approved the final manuscript.
